# Surface guided ring gantry radiotherapy in deep inspiration breath hold for breast cancer patients

**DOI:** 10.1002/acm2.14463

**Published:** 2024-08-13

**Authors:** Mustafa Kadhim, André Haraldsson, Malin Kügele, Hedda Enocson, Sven Bäck, Sofie Ceberg

**Affiliations:** ^1^ Department of Medical Radiation Physics Lund University Lund Sweden; ^2^ Radiation Physics Department of Hematology, Oncology, and Radiation Physics Skåne University Hospital Lund Sweden

**Keywords:** DIBH, SGRT, tomotherapy, VitalHold

## Abstract

**Purpose:**

This study investigated the use of surface guided radiotherapy (SGRT) in combination with a tomotherapy treatment mode using discrete delivery angles for deep inspiration breath hold (DIBH) treatments of breast cancer (bc). We aimed to assess the feasibility and dosimetric advantages of this approach.

**Materials and methods:**

We evaluated camera occlusion in the Radixact treatment system bore and the stability of DIBH signals during couch movement. The SGRT system's ability to maintain signal and surface image accuracy was analyzed at different depths within the bore. Dosimetric parameters were compared and measured for 20 left‐sided bc patients receiving TomoDirect (TD) tangential radiotherapy in both DIBH and free breathing (FB).

**Results:**

The SGRT system maintained surface coverage and precise DIBH‐signal at depths up to 40 cm beyond the treatment center. Camera occlusion occurred in the clavicular and neck regions due to the patient's morphology and gantry geometry. Nonetheless, the system accurately detected respiratory motion for all measurements. The DIBH plans significantly (*p* < 0.001) reduced mean heart and left anterior descending artery (LAD) radiation doses by up to 40%, with a 50% reduction in near‐maximum heart and LAD doses, respectively. No significant dosimetric differences between DIBH and FB were observed in other investigated parameters and volumes.

**Conclusions:**

Camera occlusion and couch movement minimally impacted the real‐time surface image accuracy needed for DIBH treatments of bc. DIBH reduced heart and LAD radiation doses significantly compared to FB, indicating the feasibility and dosimetric benefits of combining these modalities.

## 1 INTRODUCTION

The combination of a surface guided radiotherapy (SGRT) system and ring gantry tomotherapy treatment systems was released just recently. Based on our experience with such a system combination, we highlight the need for a thorough investigation of both the system's compatibility and the dosimetric benefits of using DIBH tomotherapy for breast cancer (bc) patients. SGRT can offer simultaneous real‐time tracking of the patient's intrafractional motion,^1^ by reconstructing a three‐dimensional (3D) surface of the patient from projected and reflected structured light based on the patient's surface.^2^ The SGRT technology can rely on different optical surface scanning (OSS) techniques such as structured light, stereovision, laser projection, time‐of‐flight, or thermal information to reconstruct 3D surface images through the principle of triangulation.^3^, ^4^ These systems commonly consist of three cameras to achieve optimal surface coverage of the patients^5^ allowing for 6 degrees of freedom (DoF) surface monitoring. The surface information can be used both during patient setup and during treatment delivery to activate and hold the radiation delivery.

SGRT has been mainly adopted for C‐arm linacs for patient positioning, real‐time motion monitoring, and DIBH treatments.^6^ However, SGRT is also available for patient positioning for ring gantry linacs,^7^, ^8^, ^9^ with patient setup outside the gantry bore. During the patient setup in ring gantry systems, the full 3D surface of the patient is detected by all three cameras (separated by a certain angle and distance). However, inside the bore, only the primary middle camera monitors the patient as the two side cameras are occluded by the gantry. Thus, inside the bore, the patient surface images will be reconstructed using one camera only, causing a loss of surface coverage of anatomical areas shadowed by the camera due to the gantry design and patient morphology.^7^ Optical conditions inside the bore might also affect the surface image quality. The surface image quality depends on the camera settings, surface color, and surface topography.^10^, ^11^ Furthermore, the single camera limitation affects the size of the field of view (FOV) needed to detect accurate DIBH‐signal throughout the treatment.

A well‐known challenge in bc radiotherapy is minimizing the radiation dose to the healthy tissues surrounding the treated volume, without compromising the homogeneous high dose to the tumor. The risk of major coronary and pulmonary events rises with the absorbed radiation dose to the heart and lungs.^12^, ^13^ DIBH can be implemented to potentially reduce the mean absorbed doses to the heart and ipsilateral lung while preserving sufficient dose coverage of the target.^14^ DIBH enables the separation of healthy organs at risk (OARs), like the heart, from the chest wall (location of target) while reducing intrafractional motion effects.^15^ SGRT can be utilized to deliver accurate DIBH treatments.^16^, ^17^, ^18^, ^19^

Another approach to further reduce the dose of OARs, is utilizing an intensity‐modulated radiotherapy (IMRT) fixed‐angle beam technique that delivers radiation during a constant couch movement and binary multileaf collimator (MLC) motion.^20^ Previous publications indicated dosimetric sparing of the heart and ipsilateral lung with sufficient coverage of the planning target volume (PTV).^21^, ^22^, ^23^ [20]Moreover, the IMRT technique offers fixed or dynamic jaw modes during treatment delivery, enabling MLC adjustment based on the tumor volume in the superior–inferior (SI) directions.^24^ The tomotherapy system is also equipped with a kilovoltage computed tomography (kV‐CT) imaging modality enabling high resolution imaging to reduce positioning uncertainty and inspect anatomical changes of treated regions.

Thus, our aim was to investigate the compatibility, feasibility, and advantage of combining DIBH, SGRT, and fixed‐angle tomotherapy on a ring gantry system for the treatment of bc patients.

## 2 MATERIALS AND METHODS

### 2.1 Patients' selection and delineation

A total of 20 left‐sided bc patients who received tangential radiation treatment after breast‐conserving surgery were selected. The patients were treated during year 2015−2016. The median (range) age was 59 years (45–77 years). All structures were delineated in both DIBH and free breathing (FB) CT scans by the same radiation oncologist. The delineation of structures is well described in a previously published work.^2^ No delineations of the spinal cord and contralateral breast and lung were made. As for the volunteers to in‐bore surface scanning, the number of volunteers was 4, and the median age was 29 years (27–32 years).

### 2.2 Ethical consideration and consent

The use of radiotherapy CT images for retrospective research has been approved by the Regional Ethical Review Board in Lund (No. 2013/742). All volunteers gave consent to participate in this study.

### 2.3 Computed tomography

The patients underwent CT scans in head‐first supine position in both FB and DIBH with both arms raised above the head. The CT images were acquired with a slice thickness of 3.0 mm using a Siemens Somatom Definition AS plus (Siemens Medical Solutions, Erlangen, Germany). For the DIBH scans, a Sentinel (C‐RAD Positioning AB, Uppsala, Sweden) surface scanning system was used to select a circular region of interest (DIBH‐ROI) with a diameter of 4.0 cm positioned above the xiphoid process. The ROI monitors the breathing amplitude in the anterior–posterior direction.

### 2.4 Surface guided tomotherapy

**FIGURE 1:**
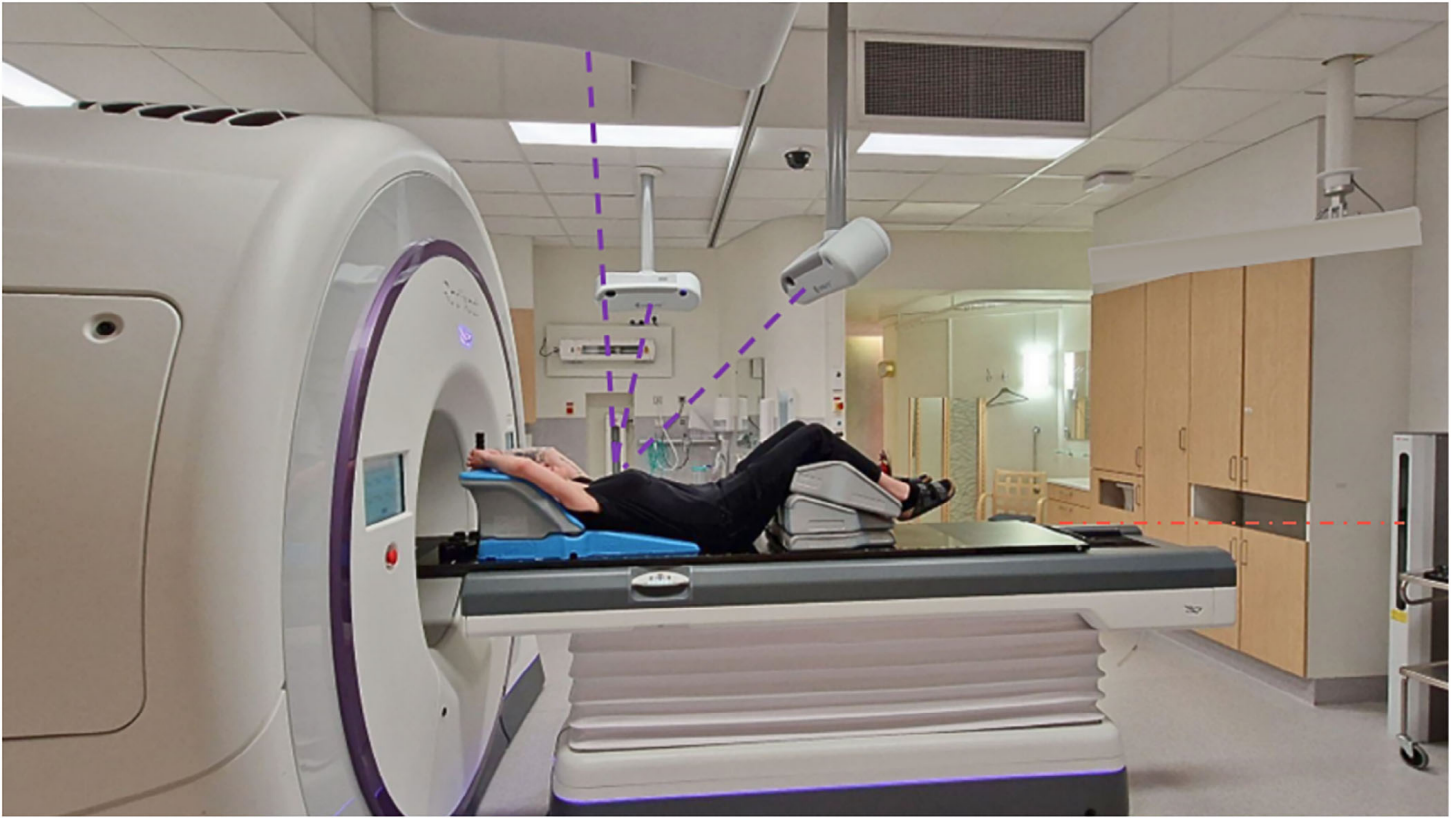
The SGRT system setup for the tomotherapy system and a healthy volunteer positioned on the couch for SGRT measurements. A laser projector device (red dashed line) is located at the end of the couch to track the position of the couch in real‐time and communicate with the main (middle) camera. The SGRT cameras (purple dashed lines) are at 120^o^ angles from each other, tilted 36^o^, and mounted 1.10 m in height (from treatment iso‐center) and 1.70 m outside the treatment iso‐center. SGRT, surface guided radiotherapy.

Our SGRT system consists of a three‐camera Catalyst^+^HD (C‐RAD Positioning AB, Uppsala, Sweden) solution with cameras installed at 120^°^ apart from the iso‐center, tilted 36^°^, mounted 1.10 m in height and located 1.70 m outside the Radixact (Accuray, Madison, USA) tomotherapy system treatment iso‐center shown in Figure 1. The middle camera was selected as the primary camera. A laser projector located at the end of the couch was used to connect to the camera system for readjustment of camera settings based on couch position. To investigate the surface coverage at relevant depths into the bore, a retrospective analysis of 4000 clinically approved bc treatment plans at our clinic from 2018−2023 was conducted to study treatment field margins in SI direction. The surface coverage by the OSS was then investigated at relevant and extreme depths into the bore to evaluate the limitation of the system.

Furthermore, the volunteers with various body contours were positioned in the Radixact using the Catalyst^+^HD. The immobilization used (Figure 1) was the all‐in‐one (AIO) Solution 3.0. (Orfit, Wijnegem, Belgium). The positioning was performed at the virtual iso‐center located 70 cm longitudinal outside the bore. Camera properties such as scan volume, integration time, and camera gain were optimized for each volunteer. The setup parameters were based on a standard tangential bc patient plan with a PTV position 4.0 cm anterior to the treatment iso‐center. Next, a reference surface was acquired to enable real‐time monitoring of each volunteer´s position along with a DIBH breathing curve. The surface coverage and DIBH‐signal were examined at the treatment iso‐center (70 cm into the bore). Later, the couch was shifted longitudinally in incremental steps of + 2.0 cm from the iso‐center into the bore until the DIBH‐signal became undetectable by the SGRT system. The distances were compared with the used field margins since 2018 for surface coverage and DIBH‐signal evaluation.

To investigate the reproducibility and drift of the DIBH signal during constant couch movement, a HexaMotion (ScandiDos, Uppsala, Sweden) platform was used to simulate respiratory motion for both FB and DIBH. An extended arm attached to the HexaMotion allowed for respiratory movement solely in the AP direction. A graph paper (5 mm resolution) was attached to the arm. A DIBH‐ROI of 4.0 cm in diameter was projected on the paper for readout of any induced DIBH‐ROI drift. The couch speeds were obtained from the treatment planning system (TPS) for each beam set. The simulated breathing curves in the HexaMotion were compared with the curves detected by the OSS to evaluate the SGRT system's accuracy. Currently, a connection between the SGRT system and the Radixact is unavailable. Hence, manual intervention was required for a beam‐on and beam‐off during treatment delivery.

### 2.5 Treatment planning

**FIGURE 2:**
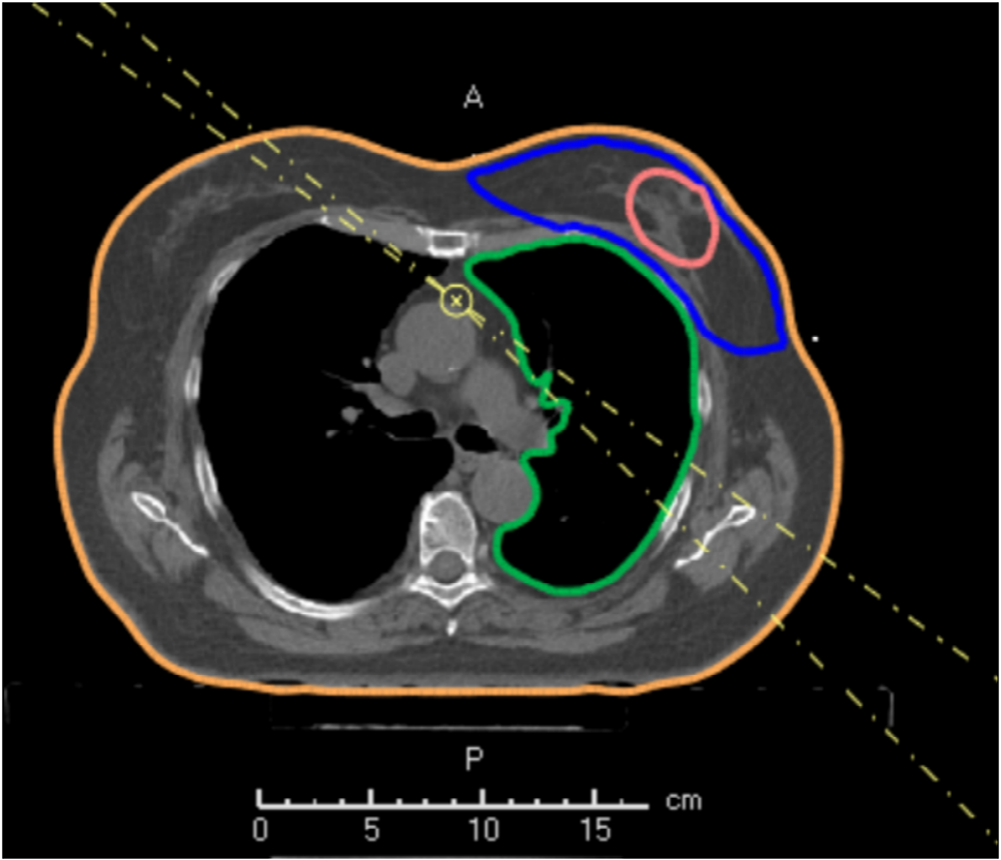
View of some of the delineated structures (PTV in blue, CTV in pink, ipsilateral lung in green, and body in orange) in a DIBH plan and a standard 4 tangential beam setup (dashed lines) used for fixed‐angle tomotherapy IMRT planning of whole breast irradiation. The delineated LAD and heart are not visible in this specific slice. DIBH, deep inspiration breath hold; IMRT, intensity‐modulated radiotherapy; LAD, left anterior descending artery, PTV, planning target volume.

The TD treatment plans were generated in the RayStation (RS) 11A (RaySearch Laboratories, Stockholm, Sweden) TPS. A total of 40 treatment plans (20 in FB and 20 in DIBH) with 4 tangential 6 MV beams were created (Figure 2). The prescribed dose to target was 50 Gy in 25 fractions normalized to the mean dose to PTV. We used dynamic jaw mode with a field width of 5.04 cm. The pitch, modulation factor, and max delivery time factor for each beam set varied in range to obtain the most optimal plan for each patient. The median (range) pitch, modulation factor, and max delivery time were 0.38 (0.15–0.59), 1.31 (1.13–1.65), and 1.30 (1.30–1.50), respectively. A robustness optimization was applied with a 1.5 cm movement span on the anterior and left sides. The beam‐on time per treatment field was optimized using the modulation features in the RS TPS.

During the optimization process, a ring structure (z_ring) of size 3.0 cm outward and 0.0 cm inward was created based on the PTV to create a dose drop‐off region. A dose calculation grid resolution of 0.25 cm was used for the final dose calculation. We followed the clinical criteria from the national guidelines of the Swedish Breast Cancer Group^25^ presented in Table 1. As for the OAR, the doses were kept as low as possible until no further improvement without compromising the PTV coverage could be achieved.

**TABLE 1:** The clinical structures and constraints to be satisfied for each treatment plan according to guidelines.^25^

Structure	Constrain
**PTV**	At least 50 Gy average dose
**CTV‐T**	At least 100% volume at 47.5 Gy dose
**CTV‐T**	At least 50 Gy average dose
**PTV**	At least 98% volume at 46.5 Gy dose
**Heart**	At most 5 Gy average dose
**Lung**	At most 20% volume at 20.0 Gy dose
**Lung**	At most 10 Gy average dose
**PTV**	At most 20% volume at 52.5 Gy dose
**PTV**	At least 90% at 46.5 Gy dose

*Note*: The prescribed dose to target was 50 Gy in 25 fractions normalized to the mean dose to PTV.

Abbreviation: PTV, planning target volume.

### 2.6 Quality assurance

To evaluate the dose distribution and plan quality, we performed plan quality assurance (QA) with the Delta4 (ScandiDos, Uppsala, Sweden) phantom, and evaluated the gamma pass rate (GPR). The selected pass rate criteria were set to 90% with a gamma (global gamma) index ≤ 1.0, a max dose deviation ± 3%, a max spatial deviation ± 2.0 mm, and a threshold of 15% of maximum dose. This was in concordance with our clinical criteria. The mean dose to the heart (D_mean, heart_), LAD (D_mean, LAD_), ipsilateral lung (D_mean, Lung_), the dose received by 2.0% of the heart volume (D_2%, heart_), the dose received by 2.0% of the LAD volume LAD (D_2%, LAD_), the percent of the ipsilateral lung volume receiving 20 Gy (V_20Gy, Lung_) and the dose received by 98% of the PTV volume (D_98%, PTV_) were acquired from the RS TPS. Both the homogeneity index (HI) and the conformity index (CI) were calculated in the TPS, where HI was defined as the ratio between D_95%_ and D_5%_, and CI was defined as the ratio of the PTV volume covered by the iso‐dose with the total iso‐dose volume. The selected iso‐dose level was 93% of the prescribed dose.

### 2.7 Statistics

To investigate any statistically significant difference between the DIBH and FB dose parameters, Two‐sided paired Wilcoxon tests were performed with a significance level of 0.05 (*p* < 0.05) on the selected dose parameters in the DIBH and FB treatment plans. The tests were performed using Python 3.9.15 and the statistics module *Scipy.stats*.

## 3 RESULTS

### 3.1 Surface coverage and DIBH‐signal stability

**FIGURE 3:**
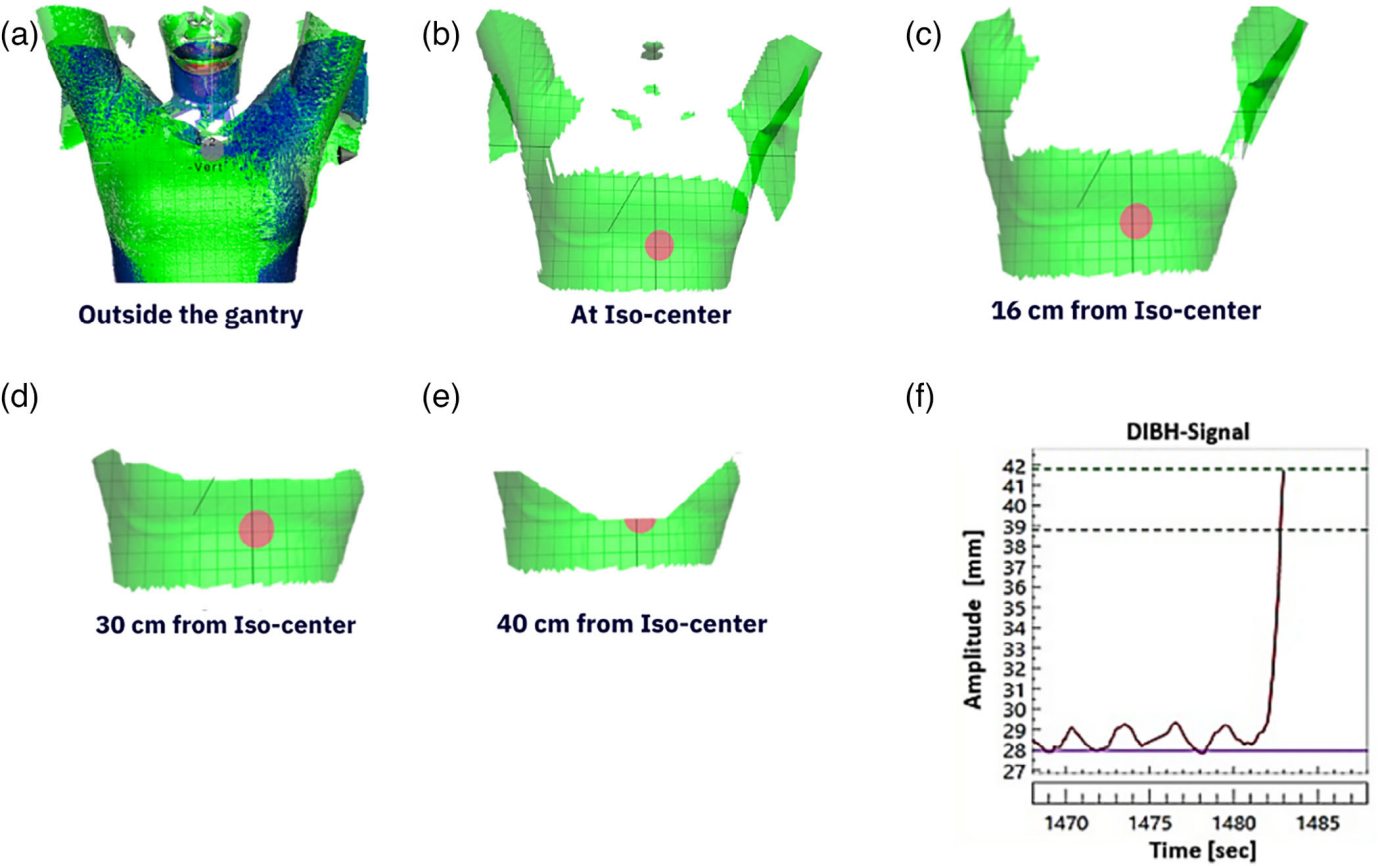
The reduction in surface image coverage due to camera occlusion and surface morphology at various positions from the treatment iso‐center of treatment system. The vertical position was 4.0 cm above the treatment iso‐center. (a) surface coverage at the virtual iso‐center, (b) at the treatment iso‐center, (c), (d), and (e) illustrates the surface coverage at 16, 30, and 40 cm beyond the treatment iso‐center. (f) a measured cut‐off in the DIBH‐signal 40.0 cm beyond the iso‐center for this specific volunteer. DIBH, deep inspiration breath hold.

Based on the collected surfaces and DIBH‐signals of the volunteers, the median (range) distance beyond the treatment iso‐center where signal loss and insufficient surface coverage were observed was 43 cm (40–46 cm) and 34 cm (31–40 cm), respectively. The values varied due to the volunteers’ different morphologies (Figure 3). A signal cut‐off can be observed when the couch is located deep into the gantry boar due to camera occlusion as shown in Figure 3f).

**FIGURE 4:**
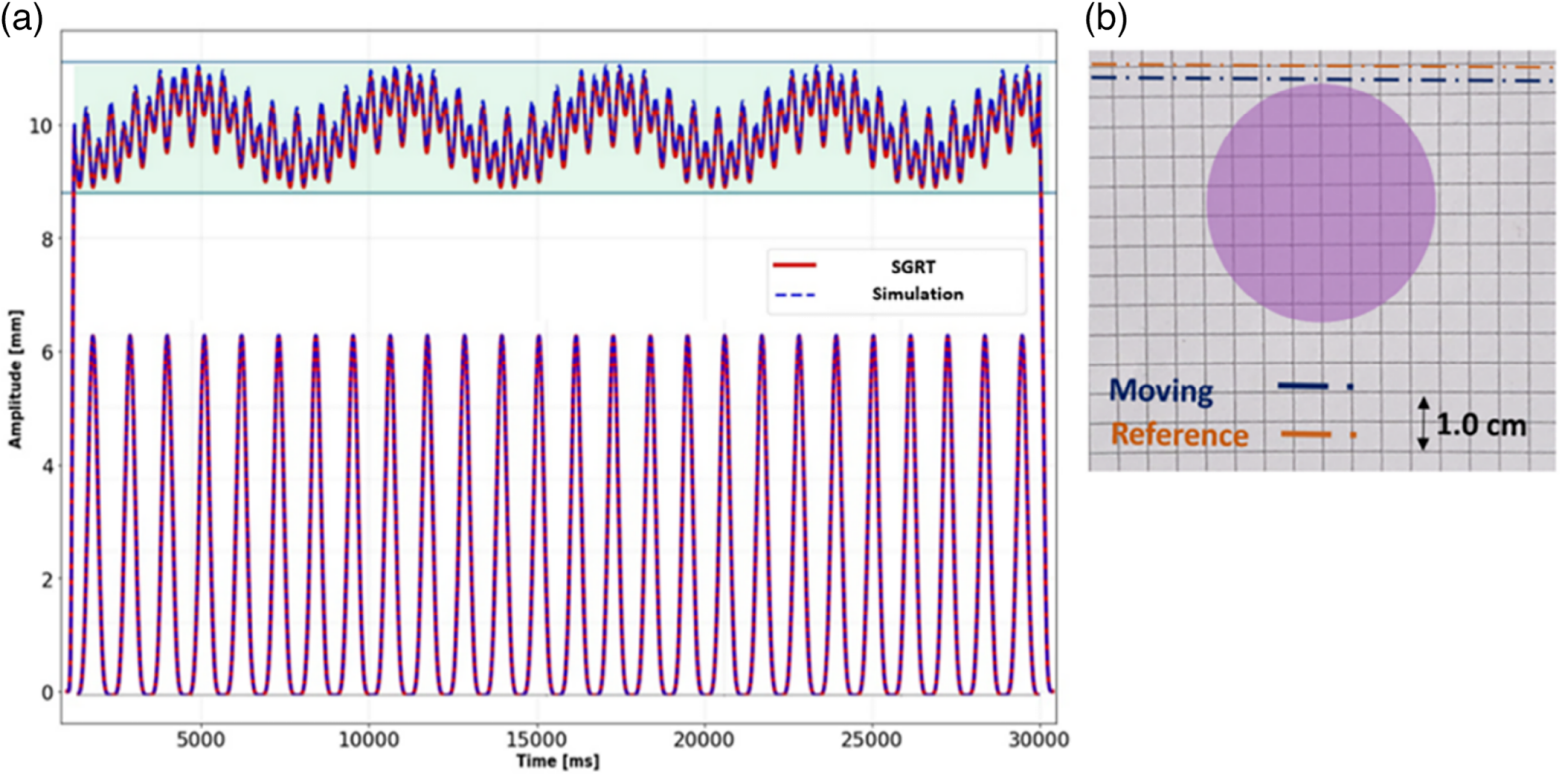
(a) The respiratory signal reproducibility by the SGRT system (red line) based on the motion of the simulation phantom (blue dashed line) for FB and DIBH. The green area represents a 3.0 mm DIBH gating window. An accurate representation of the simulated motion was observed. (b) The DIBH‐ROI (purple circle) and the detected drift of the DIBH‐ROI (blue dotted line) from the reference (orange dotted line) for clinically relevant couch speeds. The drift was less than 3 mm on the graph paper which resulted in DIBH‐signal error of less than 0.5 mm in breathing amplitude. DIBH, deep inspiration breath hold; DIBH‐ROI, deep inspiration breath hold‐region of interest; FB, free breathing; SGRT, surface guided radiotherapy.

Based on the retrospective analysis of more than 4000 clinical bc plans, the largest field size margin in SI direction was 32 cm. Approximately 80% of all patients had field size margins less than 22 cm in the SI direction. The DIBH‐ROI was able to follow the surfaces and graph paper for a median (range) couch speed of 0.68 cm/s (0.59–0.73). The speeds are comparable to clinically relevant treatment delivery couch speeds. For such speeds, the maximum induced drift was less than 3 mm (Figure 4b), which resulted in a DIBH‐signal error of less than 0.5 mm in the breathing amplitude. The detected respiratory signals by the OSS for both FB and DIBH accurately reproduced the simulated respiratory signals (Figure 4a).

### 3.2 Treatment plans quality

The DIBH plans showed a significant reduction of dose to the heart and LAD without compromising the coverage of PTV (Table 2). No significant difference between the DIBH and FB plans was observed for the **PTV_D98%_, D_mean, lung,_ V_20Gy, lung,_ PTV_HI_,** and **PTV_CI_**. Overall, the patients had lower near‐maximum dose to the heart (**D_2%, heart_**) and LAD (**D_2%, LAD_**) with DIBH in comparison to FB, where the median (range) reduction was 3.3 Gy (0.5–12.3 Gy) (*p* < 0.001) and 9.0 Gy (1.1–10.4 Gy) (*p* < 0.001) to the heart and LAD, respectively.

**TABLE 2:** The dosimetric parameters (median [range]) for both the FB and DIBH TD treatment plans.

	FB	DIBH	*p*
**PTV_D98%_ (Gy)**	**47.2 (46.2–48.0)**	**47.3 (46.6–48.9)**	**0.28**
**D_mean, heart_ (Gy)**	**1.1 (0.5–1.8)**	**0.8 (0.3–1.4)**	**< 0.001** ^*^
**D_2%, heart_ (Gy)**	**6.4 (1.6–18.8)**	**3.2 (1.1–6.4)**	**<0.001** ^*^
**D_mean, LAD_ (Gy)**	**4.8 (1.6–10.3)**	**2.8 (1.1–6.6)**	**<0.001** ^*^
**D_2%, LAD_ (Gy)**	**17.0 (3.2–35.2)**	**8.9 (2.1–25.2)**	**<0.001** ^*^
**D_mean, lung_ (Gy)**	**5.0 (3.3–7.3)**	**5.2 (3.1–7.7)**	**0.68**
**V_20Gy, lung_ (%)**	**0.1 (0.0–0.2)**	**0.1 (0.0–0.2)**	**0.40**
**PTV_HI_**	**0.9 (0.4–1.0)**	**0.9 (0.9–1.0)**	**0.74**
**PTV_CI_**	**0.7 (0.5–0.9)**	**0.7 (0.4–0.8)**	**0.82**

Abbreviations: DIBH, deep inspiration breath hold; FB, free breathing; PTV, planning target volume.

* Statistically significant difference (*p* < 0.05).

The median mean heart and LAD doses were reduced from 1.1 Gy (0.5–1.8 Gy) to 0.8 Gy (0.3–1.4 Gy) (*p* < 0.001) and 4.8 Gy (1.6–10.3 Gy) to 2.8 Gy (1.1–6.6 Gy) (*p* < 0.001) with DIBH compared to FB. The median treatment delivery time of each treatment beam of the DIBH and FB plans was 36 s (25–37 s) and 34 s (23–37 s), respectively. The mean (SD) GPR obtained from the respiratory simulation measurements of the DIBH and FB plans was 98.8% (± 2%) and 99.2% (± 3%) correspondingly, for pass rate criteria of 90% with a global gamma index ≤ 1.0, max dose deviation ± 3%, a max spatial deviation ± 2.0 mm, and a threshold 15% of maximum dose.

## 4 DISCUSSION AND CONCLUSION

In this study, we showed for the first time (1) the feasibility of using SGRT in combination with fixed‐angle IMRT tomotherapy on a ring gantry system for DIBH treatments and (2) simulated dosimetric advantages of such an approach for bc patients.

The OSS accurately reproduced the simulated respiratory motions in the ring gantry bore, while maintaining optimal camera settings regardless of the couch location in the bore. It also limited the DIBH‐ROI drifting during constant couch motion. The OSS efficiency was minimally hampered by the camera occlusion at the treatment iso‐center for all volunteers.

An optimization of the camera settings, position, tilt angles, and patient positioning angle (tilted more forward) could improve the surface coverage in ring gantry systems. A ring‐mounted SGRT camera solution or OSS system positioned both at the front and the back of the gantry has been investigated by other authors for other ring gantry systems and showed reliable results.^8^, ^26^, ^27^ The ring‐mounted gantry solution seems to be the least hampered SGRT solution by camera occlusion. Nonetheless, these previously published results concerned systems of larger bore diameter and less bore depth than our system. Our system's bore diameter and depth were 196.3 and 85.0 cm, respectively. Furthermore, the vertical couch position can influence the range of FOV of the primary camera. The higher the couch was located inside the bore, the more occlusion around the neck and chest was observed. Consequently, an adjustment of the treatment plan iso‐center should be considered during treatment planning to ensure optimal surface coverage based on the patient morphology and size of PTV. From the retrospective analysis of clinical bc plans, a rare patient case with a large PTV (29 cm) and field size margin (32 cm) in the SI direction was found in 2018. A patient with such a case treated today with tomotherapy would unlikely hamper the efficiency and accuracy of the OSS system as it demonstrated sufficient coverage of the treated area at such depths into the bore (+16.0 cm from treatment iso‐center, considering having the plan iso‐center at middle of PTV along the longitudinal direction).

An integration of a tomotherapy system with an SGRT is highly desirable to enable accurate delivery of DIBH treatments. The kV‐CT scans can be acquired in a single breath hold as opposed to the need of multiple breath holds on the conventional C‐arm linacs.^28^ This enables a quick and reliable verification of the patient anatomy and positioning prior to treatment delivery. Additionally, SGRT can facilitate positioning the patient and reduce the on‐couch time.^9^ The ability of OSS systems to track the couch movement during treatment is crucial in ring gantry systems to maintain a sufficient coverage of the patient surface, limit the DIBH‐ROI drift, and achieve an accurate DIBH‐signal. The OSS readjusts the camera settings and angles based on the couch position to compensate for the signal loss due to the gantry geometry. The coach speed had low impact on the accuracy of the OSS as the fastest couch shifts (during setup only, not relevant for treatment delivery) induced an error less than 0.5 mm in the DIBH‐signal.

For DIBH treatments of bc, short delivery times are of importance to reduce the burden and the number of breath holds required for each session. In our clinic, we instruct the bc patients to keep each breath hold less than 20 s. For conventional treatment plans delivered with the three‐dimensional conformal radiation therapy (3D‐CRT) technique at our clinic, a single beam delivery requires 1−3 breath holds (depending on the number of monitor units (MU) and patient breathing capacity). Based on the obtained delivery times for DIBH and FB with tomotherapy plans, we found the number of breath holds to be comparable to clinically implemented 3D‐CRT treatments of bc with similar number of beams.

Although our investigation focused on the tangential treatment of bc, it is worth mentioning that the camera occlusion might give rise to challenges for the treatment of bc with lymph node involvement. In which the surface image coverage of the clavicular and neck regions risk to be hampered by surface shadowing.

The SGRT technology plays an important role at our clinic in a variety of clinical tomotherapy applications such as the treatment of total marrow irradiation (TMI), total skin irradiation (TSI), and total body irradiation (TBI).

In conclusion, our work has demonstrated the feasibility and advantage of using SGRT, DIBH, and fixed‐angle IMRT tomotherapy for the treatment of bc patients.

## AUTHOR CONTRIBUTIONS

All authors contributed in the design, data collection, manuscript preparation, and its final review and editing. Mustafa Kadhim: Writing and drafting the work, data acquisition, data analysis, and data interpretation. André Haraldsson: Supervision, revising for intellectual content and final approval of the version to be published. Malin Kügele: Supervision, revising for intellectual content, and final approval of the version to be published. Hedda Enocson: Revising of methodology and interpretation of data. Sven Bäck: Supervision, revising for intellectual content, and final approval of the version to be published. Sofie Ceberg: Supervision, project administration, critical revising for intellectual content, and final approval of the version to be published.

## CONFLICT OF INTEREST STATEMENT

Our research group has an ongoing research agreement with Accuray Inc. and C‐RAD Positioning AB, which includes funding. The authors alone are responsible for the content, analysis, and writing of this work. All authors approved the final manuscript and declared that they have no potential conflicts of interest in this work.

## ETHICAL STATEMENT

The use of radiotherapy CT images for retrospective research has been approved by the Regional Ethical Review Board in Lund, Sweden (No. 2013/742). All healthy volunteers gave consent to participate in this study.

## References

[1] Laaksomaa M , Sarudis S , Rossi M , et al. AlignRT^®^ and Catalyst™ in whole‐breast radiotherapy with DIBH: is IGRT still needed? J Appl Clin Med Phys. 2019;20(3):97‐104. doi:10.1002/acm2.12553 PMC6414178 30861276

[2] Kügele M , Edvardsson A , Berg L , Alkner S , Andersson Ljus C , Ceberg S . Dosimetric effects of intrafractional isocenter variation during deep inspiration breath‐hold for breast cancer patients using surface‐guided radiotherapy. J Appl Clin Med Phys. 2018;19(1):25‐38. doi:10.1002/acm2.12214 29139223 PMC5768000

[3] Freislederer P , Kügele M , Öllers M , et al. Recent advances in surface guided radiation therapy. Radiat Oncol. 2020;15(1):187. doi:10.1186/s13014-020-01629-w 32736570 PMC7393906

[4] Brahme A , Nyman P , Skatt B . 4D laser camera for accurate patient positioning, collision avoidance, image fusion and adaptive approaches during diagnostic and therapeutic procedures. Med Phys. 2008;35(5):1670‐1681. doi:10.1118/1.2889720 18561642

[5] Al‐Hallaq HA , Cerviño L , Gutierrez AN , et al. AAPM task group report 302: surface‐guided radiotherapy. Med Phys. 2022;49(4):e82‐e112. doi:10.1002/mp.15532 35179229 PMC9314008

[6] Batista V , Gober M , Moura F , et al. Surface guided radiation therapy: an international survey on current clinical practice. Tech Innov Patient Support Radiat Oncol. 2022;22:1‐8. doi:10.1016/J.TIPSRO.2022.03.003 35402740 PMC8984757

[7] Crop F , Laffarguette J , Achag I , et al. Evaluation of surface image guidance and deep inspiration breath hold technique for breast treatments with Halcyon. Phys Med. 2023;108:102564. doi:10.1016/j.ejmp.2023.102564 36989980

[8] Nguyen D , Reinoso R , Farah J , et al. Reproducibility of surface‐based deep inspiration breath‐hold technique for lung stereotactic body radiotherapy on a closed‐bore gantry linac. Phys Imaging Radiat Oncol. 2023;26:100448. doi:10.1016/j.phro.2023.100448 37252251 PMC10213090

[9] Haraldsson A , Ceberg S , Ceberg C , Bäck S , Engelholm S , Engström PE . Surface‐guided tomotherapy improves positioning and reduces treatment time: a retrospective analysis of 16 835 treatment fractions. J Appl Clin Med Phys. 2020;21(8):139‐148. doi:10.1002/acm2.12936 PMC7484821 32592288

[10] Peng H , Jin F , Li C , et al. The impacts of colors on the catalyst HD system: gains, integral times, and setups in radiotherapy. J Radiat Res Appl Sci. 2022;15(4):100485. doi:10.1016/j.jrras.2022.100485

[11] Naidoo W , Leech M . Feasibility of surface guided radiotherapy for patient positioning in breast radiotherapy versus conventional tattoo‐based setups—a systematic review. Tech Innov Patient Support Radiat Oncol. 2022;22:39‐49. doi:10.1016/j.tipsro.2022.03.001 35481261 PMC9035716

[12] Darby SC , McGale P , Taylor CW , Peto R . Long‐term mortality from heart disease and lung cancer after radiotherapy for early breast cancer: prospective cohort study of about 300,000 women in US SEER cancer registries. Lancet Oncol. 2005;6(8):557‐565. doi:10.1016/S1470-2045(05)70251-5 16054566

[13] Darby SC , Ewertz M , McGale P , et al. Risk of ischemic heart disease in women after radiotherapy for breast cancer. N Engl J Med. 2013;368(11):987‐998. doi:10.1056/NEJMoa1209825 23484825

[14] Swanson T , Grills IS , Ye H , et al. Six‐year experience routinely using moderate deep inspiration breath‐hold for the reduction of cardiac dose in left‐sided breast irradiation for patients with early‐stage or locally advanced breast cancer. Am J Clin Oncol. 2013;36(1):24‐30. doi:10.1097/COC.0b013e31823fe481 22270108 PMC3375337

[15] Boda‐Heggemann J , Knopf A‐C , Simeonova‐Chergou A , et al. Deep inspiration breath hold—based radiation therapy: a clinical review. Int J Radiat Oncol Biol Phys. 2016;94(3):478‐492. doi:10.1016/j.ijrobp.2015.11.049 26867877

[16] Kügele M , Mannerberg A , Nørring Bekke S , et al. Surface guided radiotherapy (SGRT) improves breast cancer patient setup accuracy. J Appl Clin Med Phys. 2019;20(9):61‐68. doi:10.1002/acm2.12700 31478615 PMC6753725

[17] Svestad JG , Heydari M , Mikalsen SG , Flote VG , Nordby F , Hellebust TP . Surface‐guided positioning eliminates the need for skin markers in radiotherapy of right sided breast cancer: a single center randomized crossover trial. Radiother Oncol. 2022;177:46‐52. doi:10.1016/J.RADONC.2022.10.017 36309152

[18] Hoisak JDP , Pawlicki T . The role of optical surface imaging systems in radiation therapy. Semin Radiat Oncol. 2018;28(3):185‐193. doi:10.1016/J.SEMRADONC.2018.02.003 29933878

[19] Padilla L , Havnen‐Smith A , Cerviño L , Al‐Hallaq HA . A survey of surface imaging use in radiation oncology in the United States. J Appl Clin Med Phys. 2019;20(12):70‐77. doi:10.1002/ACM2.12762 PMC6909172 31743588

[20] Nobnop W , Phakoetsuk P , Chitapanarux I , Tippanya D , Khamchompoo D . Dosimetric comparison of TomoDirect, helical tomotherapy, and volumetric modulated arc therapy for postmastectomy treatment. J Appl Clin Med Phys. 2020;21(9):155‐162. doi:10.1002/acm2.12989 PMC7497934 32715634

[21] Reynders T , Tournel K , De Coninck P , et al. Dosimetric assessment of static and helical TomoTherapy in the clinical implementation of breast cancer treatments. Radiother Oncol. 2009;93(1):71‐79. doi:10.1016/j.radonc.2009.07.005 19682758

[22] Schubert L , Gondi V , Sengbusch ER , et al. Dosimetric comparison of left‐sided whole breast irradiation with 3DCRT, forward‐planned IMRT, inverse‐planned IMRT, helical tomotherapy, and topotherapy. Radiother Oncol. 2011;100:241‐246. doi:10.1016/j.radonc.2011.01.004 21316783

[23] Qi XS , Liu TX , Liu AK , et al. Left‐sided breast cancer irradiation using rotational and fixed‐field radiotherapy. Med Dosim. 2014;39(3):227‐234. doi:10.1016/j.meddos.2014.02.005 24857697

[24] Kim H , Jung J , Jung H , et al. Comparison of jaw mode and field width for left‐breast cancer using TomoDirect three‐dimensional conformal radiation therapy: a phantom study. Healthcare. 2022;10(12):2431. doi:10.3390/HEALTHCARE10122431 36553955 PMC9777817

[25] Swedish Breast Cancer Group . 2023;Accessed January 25, 2024. doi:https://www.swebcg.se/wp‐content/uploads/2016/09/PTVmallarutankortelengagemang20141.pdf

[26] Delombaerde L , Petillion S , Weltens C , Depuydt T . Intra‐fraction motion monitoring during fast modulated radiotherapy delivery in a closed‐bore gantry linac. Phys Imaging Radiat Oncol. 2021;20:51‐55. doi:10.1016/j.phro.2021.10.005 34765749 PMC8572954

[27] Delombaerde L , Petillion S , Michiels S , Weltens C , Depuydt T . Development and accuracy evaluation of a single‐camera intra‐bore surface scanning system for radiotherapy in an O‐ring linac. Phys Imaging Radiat Oncol. 2019;11:21‐26. doi:10.1016/j.phro.2019.07.003 33458272 PMC7807582

[28] Prado A , Zucca D , De la Casa MÁ , et al. Intrafraction target shift comparison using two breath‐hold systems in lung stereotactic body radiotherapy. Phys Imaging Radiat Oncol. 2022;22:57‐62. doi:10.1016/j.phro.2022.04.004 35514526 PMC9065403

